# The Concept of Child-Centred Care in Healthcare: A Scoping Review

**DOI:** 10.3390/pediatric16010012

**Published:** 2024-02-01

**Authors:** Bernie Carter, Sarah Young, Karen Ford, Steven Campbell

**Affiliations:** 1 Faculty of Health, Social Care and Medicine, Edge Hill University, Ormskirk L39 4QP, UK; 2 Launceston Clinical School, Tasmanian School of Medicine, College of Health & Medicine, University of Tasmania, Launceston, TAS 7250, Australia; sarah.young@utas.edu.au; 3 School of Nursing, College of Health & Medicine, University of Tasmania, Hobart, TAS 7000, Australia; karen.ford@utas.edu.au (K.F.); steven.campbell@utas.edu.au (S.C.)

**Keywords:** child-centred care, agency, participation, decision making, communication, impact

## Abstract

Although child-centred care is increasingly referred to within the nursing literature, a clear definition of child-centred care and clarity around the concept is yet to be achieved. The objectives of this review were to examine the following: (1) What constitutes the concept of child-centred care in healthcare? (2) How has the concept of child-centred care developed? (3) What is the applicability of child-centred care and what are its limitations? (4) How does the concept of child-centred care benefit and inform children’s healthcare? In total, 2984 papers were imported for screening, and, following the removal of duplicates and screening, 21 papers were included in the scoping review. The findings suggest that child-centred care is an emerging, ambiguous poorly defined concept; no clear consensus exists about what constitutes child-centred care. Although it seems antithetical to argue against child-centred care, little robust evidence was identified that demonstrates the impact and benefit of child-centred care. If child-centred care is to be a sustainable, convincing model to guide practice and compete with other models of care, it needs to establish robust evidence of its effectiveness, the impact on children and their families, as well as the wider impacts on the healthcare system.

## 1. Introduction

The position of children in healthcare reflects their changing and evolving positioning in society more broadly [1]. The concept of child-centred care orientates children to a more central position within children’s healthcare, where the child is at the centre of thinking and practice [1,2,3].

The concept of child-centred care adds to the different notions or concepts of centredness used to describe the focus of healthcare in general as well as healthcare for children and young people [4]. Other facets of centredness in healthcare include person-centred (and patient-centred) care, family-centred care (FCC) and various (and perhaps confounding) combinations of these. The precise meanings of each are subject to confusion and misunderstanding as well as uncertainties. Reactions or responses to societal shifts can be seen as the drivers for these different concepts. The different terms are considered in the following section.

The term ‘person-centred care’ (PCC) evolved from the term ‘patient-centred care’ and is a loosely defined term [5,6] but typical definitions present it as a holistic approach to care that is respectful and individualised, that includes negotiation of care, choice and where persons receiving care are empowered to be involved in health decisions at the level they choose [4,7]. PCC was described in the mid-20th century when there was a call to understand the patient as a whole person. Key attributes of PCC are that patients should be treated as individuals and with respect and dignity and that their needs, wants and preferences are included in care planning [8]. In PCC, the focus is on the individual—an adult with autonomy [4]. PCC has been seen to typically, or traditionally, refer to adults rather than children, with a strong representation within the mental healthcare, older person and dementia care literature [4].

In the context of children’s healthcare, FCC recognises that children need to be cared for in the context of their family, that families are the constant in the child’s life and the family’s values and priorities should be central in the plan of care for the child [9]. The theoretical origins of FCC came from the understanding of child attachment theories and recommendations from the Platt Report [10], which also had a significant influence on changes to care of children in hospitals. Where parents were largely excluded from children’s hospital wards in the 19th and the first part of the 20th century, there have been incremental changes towards acceptance of parents, their presence and building partnerships [11,12,13].

In FCC, the family is the unit of care [14] and involves healthcare providers working in partnership with families, and the care of the child is in the context of their family [3]. In FCC, the focus is on adults—the parents and health professionals rather than the child. In FCC, parents and health professionals are the recognised active members and children and young people are allocated a more passive and less prominent role [1,15]. FCC is described as having been a preferred approach to children’s healthcare for some decades; however, complex issues have been identified that compromise the effectiveness and implementation of this model including relationship, attitudinal and resource factors [2,14].

Current constructions of childhood that lie within an emancipatory, rights-based, citizenship-oriented and participatory paradigm [16] have been major drivers in the development of the concept of child-centred care [17,18]. Child-centred care in healthcare reflects the broader societal view of children’s rights that is framed by the UN Convention on the Rights of the Child [19]. The approach recognises children as social actors both in their own right and as active participants in their care, with its theoretical origins in the new sociology of childhood [20].

Rather than a model that provides a method or recipe for achieving child centredness, child-centred care is seen as an approach or philosophy that underpins and informs children’s healthcare. The approach places children at the centre of healthcare practice and, where able, children and young people are included as active participants in their care and decision making [2,4]. Child-centred approaches recognise that children and young people experience illness and disability differently than adults and that their healthcare needs are different than those of adults [2]. The premise that the best interests of the child should be the paramount consideration underpins the approach [2]. In child-centred care, the central role of parents and families in relationships and interactions continues to be acknowledged [2,4].

The difference between child-centred care and FCC is one of emphasis based on the extent to which children’s interests are highlighted or prioritised in the planning and delivery of care [21,22]. In child-centred care, the focus is on the child in the context of their family. Child-centred care acknowledges the need to specifically focus on children and young people. It also recognises that their views and concerns are not necessarily the same as those of parents/carers or healthcare providers [2,15,23].

PCC and child-centred care have more similar attributes than child-centred care and FCC [4]. These commonalities include competence, values, own needs and active participation [4].

Although the concept of child-centred care is increasingly referred to in the healthcare literature, particularly within the nursing literature, a clear definition of child-centred care and clarity around the concept is yet to be achieved and further work is needed in developing the definition [15,24].

### Aims and Objectives

The aim of this scoping review was to identify the concept of child-centred care in healthcare, to provide clarity on the concept of child-centred care, and to evaluate its application/appropriateness.

The main objective of this review was to examine what constitutes the concept of child centred within children’s healthcare. 

Further objectives included the following:

- How has the concept of child-centred care developed?
- What is the applicability of child-centred care and what are its limitations?
- How does the concept of child-centred care benefit and inform children’s healthcare?

## 2. Methods

The scoping review protocol was published in 2018 [24]. This scoping review was designed with the intention of evaluating the concept of child-centred care in healthcare in order to achieve clarity on the concept and its applicability, benefits and potential to inform the evidence base of children’s healthcare policy and practice. To achieve this aim, a literature review method was adopted, using the Preferred Reporting Items for Systematic Reviews and Meta-analyses Extension for Scoping Reviews (PRISMA-ScR) approach [25,26,27].

As this was a scoping review, no ethics approval was required.

### 2.1. Inclusion Criteria

In a scoping review, it is important to establish inclusion criteria to determine which studies are eligible for inclusion in the review [26]. In this case, the context of included studies was those related to children and adolescents in any setting where healthcare may be provided (e.g., in-patient and out-patient settings; tertiary, secondary and primary care settings; respite and hospice settings; medical home, home-based care and school settings) [24]. The types of studies included in this review include peer-reviewed papers and opinion papers. In light of the complex nature of this scoping review that addresses the development of the concept of child-centred care, the decision was made not to include documents related to policy documents from governments, healthcare organisations, professional bodies and consumer advocacy groups.

### 2.2. Search Strategy

Several databases (CINAHL, MEDLINE, Web of Science) were searched using a combination of terms. This search strategy was developed to enable papers to be identified that related to child-centred care. The selected search terms were chosen based on our knowledge of the topic. Studies were restricted to the English language with a date range of 1990–2021. The year 1990 was chosen as the cut-off date as this is the point at which the concept of CCC in healthcare appears in the literature. Peer-reviewed literature, as well as some grey literature and dissertations, were eligible for inclusion in the study. Search terms included the following: (child+ OR adolescence+) OR (child OR children OR adolescent); adolescent health services OR child health services+ or family-centered care+ OR patient-centered care+; (child* centered OR child* centered OR child* rights OR child* perspective OR child* voice OR child* view OR child* participation OR child* involvement) OR patient autonomy OR decision making, patient+ OR decision making, family OR patient rights+; and combinations of these searches. For more details of these searches across the databases, please refer to the Supplementary Materials.

### 2.3. Screening and Eligibility

**Figure 1:**
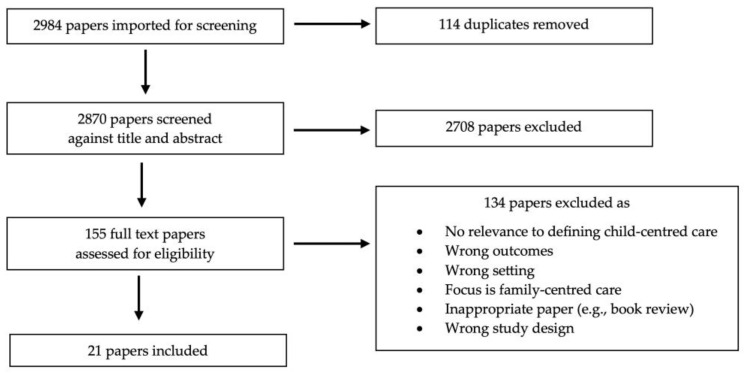
PRISMA flow diagram for the scoping review process.

From the three databases, 2984 results were retrieved and exported to Covidence^®^, and after the automatic removal of 114 duplicates, 2870 records remained. The titles and abstracts of these records were assessed against the inclusion criteria listed above. All abstracts and metadata were imported into Covidence^®^ to facilitate the implementation of the PRISMA-ScR screening approach (see Figure 1). Of the 155 papers remaining after title/abstract screening, 134 were excluded after a full-text review, leaving 21 papers for inclusion. Three authors (SY, KF, BC) participated in the abstract/title screening process, and three authors (SC, KF, BC) participated in the full-text review process. The validity of the papers included in the full-text review was assessed against the following three eligibility criteria:

- The focus of the paper was adequately on child-centred care and not FCC;
- There was sufficient content relevant to defining child-centred care on a practical or conceptual level, including papers that may not have used the term child-centred care but whose content was relevant to the germinal concept of child-centred care;
- The outcomes and setting were relevant to this scoping review.

If the three criteria above were met, the paper was retained. Figure 1 shows the flow of papers through the review process.

### 2.4. Data Extraction and Charting

For each paper, the author, year, study population, country of origin, intervention type, study aims, methodology, outcome measure(s) and important results were abstracted from the article, as per the guidance for scoping review procedures [26,28]. A summary of these findings is shown in the data extraction tables (Table 1 and Table 2), which present a summary of key data from the review.

## 3. Results

The results are presented in a narrative format. First, an overview of the papers is presented, followed by five themes integral to the concept of child-centred care. Themes were identified using an inductive approach. Recurring concepts or elements of child-centred care in the included papers were identified by the team in collaboration and the resultant themes were determined. Individual team members then searched each of the included papers for the presence of the named theme (or associated synonyms).

### 3.1. Demographics of Included Papers

Twenty-one papers were included in the review. Of these, 10 papers were categorised as discursive [1,3,4,15,23,29,30,31,32,33] and 11 were empirical papers [34,35,36,37,38,39,40,41,42,43,44].

### 3.2. Overview of Discursive Papers

#### 3.2.1. Dates of Publication

The 10 discursive papers were published between 2011 and 2021, with most (n = 7) published between 2011 and 2018 [1,3,4,15,23,30,33] and three published in 2020–2021 [29,31,32].

#### 3.2.2. Authorship

Primarily, papers were co-authored by people from Europe (Ireland [3,4], Sweden [3,4,23], the UK [1,36], Australia [1,15,31], and New Zealand [1,30,31], with one paper each from South Africa [33] and Canada [32], and one with extensive international authorship [29].

#### 3.2.3. Discursive Focus

Most papers (n = 4) were focused on a conceptual consideration of CCC in relation to other models and focused on types of ‘centredness’ such as FCC and PCC [3,4,15,31]. Two papers were proposing or considering a merger of child-centred care with FCC with one paper proposing that the merger should be ‘family and child-centred care’ (FCCC) [30] and the other proposing a different ordering of the concepts with the child as the lead concept with the term being ‘child and family-centred care’ (CFCC) [32]. Individual papers addressed child-centred care in relation to other theories [1].or in relation to children’s rights [33] or perspectives [23]. One paper was a position statement on CCC [29].

### 3.3. Overview of Empirical Papers

#### 3.3.1. Dates of Publication

The 11 empirical papers were published between 2005 and 2022; 1 was published in 2005 [36] and then there was a gap until 2012, with 4 papers published between 2012 and 2019 [35,41,43,44] and the remaining 6 papers were published between 2020 and 2022 [34,37,38,39,40,42].

#### 3.3.2. Countries Data Generated from

Primary data was generated from five countries: Sweden (n = 4) [34,37,39,40], the UK (n = 2) [36,38], New Zealand (n = 2) [41,42], the USA (n = 1) [44] and Canada (n = 1) [35]. One paper was a systematic review in which most papers were from the UK, USA and Europe [43].

#### 3.3.3. Study Design

Most papers (n = 8) used a qualitative design [34,35,36,37,38,40,41,42]. Of these, two used a phenomenological approach [41,42], one used grounded theory [34], one used participant inquiry [36] and one used a case study [35], with the remaining three papers not stating a specific design [37,38,40].

Two papers reported using a quantitative approach [39,44]; of these, one was a feasibility study using a non-randomised quasi-experimental cluster design [39] and one was used for secondary data analysis [44].

One paper was a systematic review [43].

#### 3.3.4. Level of Child Involvement

Since the focus of this review was on child-centred care, it seemed appropriate to try and appraise the level to which work underpinning the papers involved children. To this end, we created three, arguably crude, categories that could be utilised to report the level of child involvement. The categories were ‘no involvement’ (papers in which there was no evidence of child involvement or engagement, e.g., in academic opinion pieces where the only voice is that of academics/researchers); ‘marginal involvement’ (evidence of some indirect involvement of children); and ‘authentic involvement’ (evidence of direct involvement of children, such as in research studies where children’s voices were either evident or children acted as advisors). Five papers were categorised as indicating ‘authentic involvement’ [34,36,38,39,40], four papers were categorised as ‘marginal involvement’ [35,37,41,42] and two categorised as ‘no involvement’ [43,44].

#### 3.3.5. Sample Size and Characteristics

The sample size of children ranged from 2 [35] to 785 [39], with most falling in the range of 10 [36] to 26 [38,41] children.

Samples also included other stakeholders such as parents (total n = 839) [38,44], siblings (n = 13) [38], health professionals (total n = 34) [37,38,42] and other stakeholders (n = 15) [38].

Most papers were in the range of 4–15 years [34,38,39,40,41], with one paper including a child aged 2 years [36] and one paper including a case report on a 24 year old [35].

### 3.4. Themes

**Figure 2:**
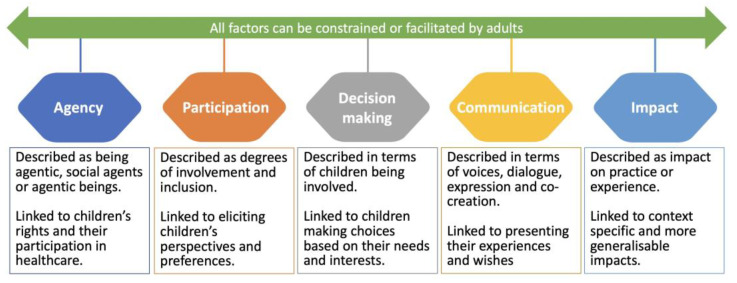
Overview of key themes.

Five themes were identified: agency, participation, impact, decision making and communication (see Figure 2).

#### 3.4.1. Agency

Of the 21 papers, 10 made direct reference to children being agentic, social agents or agentic beings [1,3,4,32,35,38,39,40,41,42]. Of these, six were empirical papers [35,38,39,40,41,42] and four were discursive [1,3,4,32]. Although one of the empirical papers mentioned agency frequently, it was not directly related to CCC but to a relational ethics framework [35].

Agency and its synonyms were linked to children’s rights [1,3,4,38]. Agency and being agentic were linked to participation in general [3,32] and, more specifically, participation in the construction of their own lives [4], in healthcare [38], health dialogues [39] and care planning [42].

Children’s agency reflects adults respecting children’s independence [1,3], competence [1], ability to construct an understanding of issues related to them [40] and acknowledgment of their experiences [3]. Agency or being agentic was linked to partnerships and/or collaborations and interconnectedness [4,41] and respect [3].

Agency was reported as being constrained or shaped by adults [1,32], as well as the exclusion from research participation through a reliance on proxies [38], or by limitations to inclusivity of existing methodologies and methods [32] and to equity of opportunity [32]. 

#### 3.4.2. Participation

Of the 21 studies, 18 referred to children’s participation. Of those 18 studies, 8 were empirical papers [34,36,37,38,39,40,41,44] and 10 were discursive [1,3,4,15,23,29,31,32,33,35]. One empirical paper identified participation solely in terms of [36] research.

The synonyms ‘involvement’ and ‘inclusion’ were also used to indicate participation. Participation as a concept is not well defined [34] but includes participation in decision making and care and eliciting children’s opinions, perspectives and preferences [3,29,34,37,38,39].

Participation was identified as essential in accordance with children’s rights, with 14 papers referring to the UN Convention on the Rights of the Child and/or national guidance [1,3,4,15,23,29,31,32,33,35,37,38,39,40].

Although a prerequisite for rights-based child-centred care, the level of participation can vary from exclusion, limited opportunities [34,35] or minor degrees of participation in more trivial matters [34], to involvement in serious subjects such as complex care [35] and end of life [37,38].

Like agency, children’s participation can be enabled or constrained by adults, both healthcare professionals and parents. Children are dependant on adults and the systems around them to ensure and optimise their participation [1,3,23,29,32,34,41].

Participation should be at a level of the child’s own choosing [32,36,40]. The child’s age, maturity and competence are factors influencing participation [1,4,23,39,41]. Children are more capable of participating in matters of concern for them than is often recognised [35].

#### 3.4.3. Decision Making

Of the 21 papers included in this review, 12 mentioned decision making in some way [1,3,4,15,23,29,34,35,38,40,41,43]. Of the empirically based papers, six referred to decision making [34,35,38,40,41,43]. And for the discursive papers, six also referred to decision making [1,3,4,15,23,29].

In terms of defining decision making, this was loosely presented and has clear links with another theme in this review: agency. For the discursive papers, there were positive statements about children being involved in decision making [34,40], clear statements about them not being involved in decision making [35,43] and calls for children to be involved in decision making [38,41]. Calls were made for children to be involved in their own decision making [34] and it was noted that children liked to participate and that they could influence their choice. However, others made claims about the exclusion of children’s voices [35] and that they are rarely involved in decision making. The assertions included the child’s needs and interests always being at the centre of care and decisions [38] and children needing to be “creators of their own healthcare experience” [41]. For the discursive papers, the divisions were not quite so clear; there were assertions for positive involvement in decision making [29,43] and participation in all aspects of healthcare delivery (including decision making) [3]. Clearer was the discussion about whether the views of parents should supersede the views of children in decision making [1,23], which was not overt in the empirical papers. Notions of dignity impinge on this, including whether dignity can be maintained if the child is not involved in the decision making [4].

#### 3.4.4. Communication

Of the 21 papers included in this review, 14 mentioned communication in some manner [1,3,4,15,23,31,34,35,36,39,40,41,43,44]. Of the empirically based papers, seven [34,35,39,40,41,43,44] referred to communication in some form. And for the discursive papers, six papers [1,3,15,23,31,36] discussed some aspects of communication.

Various terms are incorporated in this interpretation of communication [44]. For the empirical papers, these included the following: creating a communication space [34], children’s voices [35], dialogue [40], expression of views [39,40], co-creation [41], information sharing [43] and ‘sitting with’ [44]. For the discursive papers, a similar and complementary set of terms was discussed. These included encouraging dialogue [36], children representing their own experiences and wishes [3], Ubuntu-type interdependence [1] and conversations about support [31]. Having a right to participate [15] was also mentioned, including the need to consider children’s perspectives to improve the way they are treated [23].

As shown above, the terms for communication often fence around the actual term. Few directly discussed communication [15,39,40,44]. However, the other aspects cannot occur without communication with the children. It is also clear that communication is not a clearly defined aspect of child-centred care, including its key role in achieving child-centred care.

#### 3.4.5. Impact

There is limited discussion of the impacts of child-centred care on any level amongst the papers included in this review. In total, 10 of the 21 papers included mentioned the impact of CCC on some level, be it on practice, outcomes or experience [1,15,32,34,36,37,38,40,42,43]. Of these papers, three were discursive [1,15,32] and seven were empirical [34,36,37,38,40,42,43]. Of the seven empirical papers, none were quantitative studies, six were qualitative studies [34,36,37,38,40,42] and one systematic review was included [43]. There is limited evidence of broad outcome or impact assessment, as evidenced by the lack of quantitative empirical papers included in this section.

Of the ten papers that discuss the impact of child-centred care, six (five empirical [34,37,40,42,43], one discursive [1]) included a direct reference to the impact of child-centred care, but often these impacts were context specific and not generalisable [34,37,40,42]. For example, one paper [42] showed that a child-centred care model is an approach preferred by healthcare providers in an acute situation where the child has been potentially endangered by their family (in this case, non-accidental head injury of infants). Other papers made more generalisable, less context-specific conclusions [1,43]. One paper stated that child-centred care is becoming more significant in terms of shaping children’s healthcare [1], and another that child-centred care has positive impacts on children in terms of self-esteem, patient empowerment and numerous treatment outcomes [43].

One paper ([38] empirical) included more indirect reference to the impacts of child-centred care, including a discussion of potential rather than assessed impacts, and that development of a child-centred outcome measure was needed but not yet developed. One paper ([36] empirical) cited both direct and indirect impacts of child-centred care on practice. This paper included empirical findings from a child-centred intervention, but also concluded, more indirectly, that children’s perspectives should have a greater influence on future practice.

Two papers (both discursive) overtly cited a lack of evidence for the provision of child-centred care [32] and its implementation/effectiveness [15].

## 4. Discussion

In this scoping review, 21 papers were reviewed, and five themes (agency, participation, impact, decision making, and communication) were identified as being perceived and reported to be core to the concept of child-centred care in healthcare. In the discussion, these themes are considered in the context of the wider literature on children’s agency, participation, decision making and communication. The discussion also contextualises child-centred care in the wider discussions of centredness and person-centred care in healthcare. The discussion also considers the development and adoption and application (or lack thereof) of child-centred care and its limitations and benefits.

### 4.1. What Constitutes the Concept of Child-Centred Care in Healthcare?

What is clear from the review is that there is no clear consensus across the papers about what constitutes child-centred care, suggesting that it is an emerging, ambiguous and poorly defined concept. However, four interconnected concepts—agency, participation, decision making and communication—were identified or discussed to some degree in many but not all papers. These concepts are ones that typically appear in the contemporary literature about children’s positioning within society, healthcare and children’s health literacy and it would be hard to argue that any of these are unimportant. However, even when these concepts were present in the reviewed papers, they were often simply referred to rather than clearly defined, perhaps reflecting the complexity of such concepts and the fact that definitions are contested. For the most part, the papers neither state the depth or degree to which agency, participation, decision making and communication should be present nor how they can be enacted authentically to ensure that child-centred care ensues. The belief that child-centred care is important is evident in the reviewed papers and this aligns with other work that proposes that the importance of child-centred approaches to care is key to good quality care (see, for example [38]).

In the review, the agency was mostly discussed in terms of children’s rights [1,3,38] and their positioning in society, and closely linked with participation [3,4,32]. Agency is argued to result from relationships between human beings and their environment [45], which is a continuum characterised by interdependence [46]. Agency is clearly important as it requires adults to acknowledge the inherent wisdom and skills of children and young people [47], perceiving them as citizens [16]. Healthcare professionals who wish to work in child-centred care ways need to accept that children are already beings with agency who can reflect on and co-construct their worlds [48]. This means that child-centred care requires healthcare professionals and organisations to ensure that they reduce barriers to children acting agentically, and create opportunities for children to actively participate and enact their agency, such as, for example, through shared decision making [49] and participation in clinical encounters [50] and during periods of hospitalisation [51], as well as interventions focusing on health and well being [52]. However, as seen in the review, e.g. [3,34,35,37], research that specifically focuses on children’s participation in medical encounters reveals that their participation is typically marginal [53,54].

Agency and participation require the acknowledgment that children and health professionals are actors within what has been described as a networked system [55]. In a networked system, everything affects everything else, meaning that factors (in the case of our review, participation, agency, decision making and communication) are contingent on each other and competing agendas and, ultimately, interdependent [46]. Child health literacy is a field with growing momentum, and closely mirrors the core concepts of participation, agency, decision making and communication identified in our review. The current global attention being given to health literacy in general, as well as to child health literacy, may well be a driver towards achieving child-centred care.

The review found that communication was perceived to be a core element of child-centredness and that this involved creating a space for communication [34] and supporting children to be able to express their views and engage in dialogue and conversation [31,36,39,40]. Recent work addressing child-centred communication strategies aligns with findings from the review and proposes core steps (greet, engage, involve and share) upon which good communication, even in time-limited encounters, can be built [56]. Other work, albeit not expressed as overtly child centred, supports the need to actively promote communication with all children [57,58], respect children’s expertise [59] and address health literacy issues [60]. This shift toward more child-centred communication practices can be seen in the endorsement of using resources co-developed with children and young people to support communication [61]. 

Decision making was another aspect of child centredness that was identified in the review (see for example [34,38,40]. However, there is robust evidence that shared decision making is not consistently implemented, often resulting from barriers such as healthcare professionals having insufficient time, the presence of power imbalances and healthcare professionals not having the requisite skills for shared decision making [62]. To overcome such issues, strategies to promote shared decision making include the use of decision-support tools to facilitate the participation of children. However, the review also noted the tension between whether the views of parents should supersede the views of children in decision making [1,23]. The ethics of whose voice (child or parent) should hold sway and in what circumstances is complex and contextual. Yet, until children can be active participants in communication that concerns them, engagement in decision making is not possible. Research shows that children are often marginalised in triadic (child–parent–healthcare professional) clinical encounters [54] and that dialogue is often dyadic (parents–professionals) [53], resulting in exclusion of children’s perspectives.

The review revealed a lack of evidence for the impact of child-centred care and how children benefit from child-centred care. This is perhaps unsurprising considering that the more firmly embedded concept of FCC in children’s healthcare is still reported to lack robust impact evidence [15,32,63,64]. No clearly defined consensus measures to determine the impact of child-centred care were evident within the review, reflecting the lack of attention to developing measures and/or measuring the impact of child-centred care in the literature. This is somewhat at odds with what is seen in the much larger field of (primarily adult) patient-centred care, which is now widely recognised internationally as a means of delivering high-quality healthcare. A meta-narrative review of patient-centred care [65] identified 50 measurement instruments being used, albeit only 10 of these were directly measuring patient-centred care. If child-centred care is to be a sustainable and convincing model to guide practice and be able to compete with other models or frameworks of care, it needs to establish robust evidence of its effectiveness. Other facets of child health practice that are child centred, if not completely embodying child-centred care, include child-centred outcome measures and child-centred experience measures. Scott et al. [66] argue that using person-centred outcome measures in “routine paediatric care is key to child-centred quality care” (p42) but they note that implementation of and adherence to such measures is not simple and barriers exist. 

Evidence from different countries with different health systems shows that the lack of a systematic approach, at all levels in an organisation, can impede the well-integrated adoption of person-centred care [67]. Successful adoption requires the use of evidence-based knowledge, guidelines and national regulations [67]. The lack of a clear evidence base for impact and benefit, as well as a lack of guidelines and regulations, perhaps provides a rationale for why child-centred care has not, so far, been effectively adopted across healthcare systems.

### 4.2. How Has the Concept of Child-Centred Care Developed? 

It is difficult to identify whether or if child-centred care has developed over the period covered in this review, as the term has been used loosely; a lack of definitional sources lies at the root of this challenge. What is clearer is the tension between child-centred care and FCC [32]. Previously, there was no seeming questioning of the relationship between the two forms of care [32]. Now, there is greater evidence of a realisation that, while the two forms of care can be mutually supportive, they can also be at odds with each other, and the rights of children (for instance, a child’s right to be involved in decision making) might be “trumped” by parental rights [23]. There is an overlap between child-centred care and person-centred care. However, work relating to person-centred care typically focuses on adults [68] and there is the potential that person-centred approaches miss the particularities, uniqueness and changing dynamic of providing care for children. A future development proposed is melding the concept of child-centred care with FCC to become child and family-centred care (CFCC) [32], although this is likely to just blur the distinction between the two concepts and may not necessarily advance the position of children’s agency, participation and decision making. 

### 4.3. What Is the Applicability of Child-Centred Care and What Are Its Limitations? 

The applicability of child-centred care lies in its potential to create a better balance in terms of power [23], empowerment [69] agency [32,40], participation [34,35,38,39] and respect for the child [3]. In many circumstances, the evidence shows that family-centred care has essentially become parent oriented and often primarily oriented to maternal involvement [3]. Although not always talked of in terms of child-centred care, there is evidence in the other literature, that aligns with child-centred care values, such as dealing with the rights of the child and the importance of listening to the voice of the child and responding to their expressed needs [57,70,71,72]. However, ensuring that care genuinely becomes child-centred means that their voices and wishes should be given primacy wherever possible. The limitations of child-centred care are twofold: firstly, the legal rights for decision making lie with the parents [72], and secondly, the assumptions about the capacity of the child to be involved in decisions about their care, rather than be a passive receiver of care.

### 4.4. How Does the Concept of Child-Centred Care Benefit and Inform Children’s Healthcare?

A child-centred care approach has the potential, if implemented effectively, to acknowledge and reaffirm the rights of the child outlined in the United Nations Convention on the Rights of the Child (UNCRC) [19]. The approach recognises children’s rights to participate in healthcare matters and decisions about their care. The concept of child-centred care positions children in a more central orientation within healthcare, so that the focus is on the child in the context of the family rather than the other way around (where the child’s perspectives are secondary).

Considering the evidence from the person-centred care literature where benefits are considered in the wider context such as impacts on patient safety [73], rationales proposed for child-centred care need to extend to debates about wider contextual issues rather than being inwardly focused.

### 4.5. Strengths and Limitations of the Review

The strength of the review is that it focused on the literature that specifically included the term child-centred care. However, the requirement for this specific term to be used resulted in the exclusion of many papers that were inherently child-centred in spirit, but which did not refer to the term. This resulted in the inclusion of only 21 papers and, of these, 10 were discursive and most empirical papers reported using qualitative approaches. More detailed and extensive research needs to be undertaken to create a more robust knowledge base from which to argue the merits or otherwise of child-centred care. Future research needs to consider the use of quantitative methodologies to provide complementary evidence to the existing qualitative evidence.

## 5. Conclusions

The findings from the review suggest that child-centred care is an emerging, ambiguous and poorly defined concept with no clear consensus about what constitutes child-centred care. Although it seems antithetical to argue against child-centred care, little robust evidence was identified that demonstrates the impact and benefit of child-centred care. If child-centred care is to be a sustainable and convincing model to guide practice and able to compete with other models or frameworks of care such as person-centred care, it needs to establish robust evidence of its effectiveness, impact on children and their families, as well as the wider impacts (such as patient safety and cost effectiveness) on the healthcare system. 

It is difficult to identify whether or if child-centred care has developed over the period covered in this review, as the term has been used loosely; a lack of definitional sources is at the root of this challenge.

## Figures and Tables

**Table 1:** Discursive papers included in the review.

Author, Year, Country of Origin	Aim	Key Points
Al-Motlaq et al., 2021 International [29]	To create an international position statement about child and family-centred care (CFCC).	An international position statement of the INCFCC on the provision of CFCC during the era of COVID-19, as children and families are most likely to be affected due to restrictions being placed on family presence and involvement in the care of their children.
Coyne et al., 2016 Ireland, Sweden [3]	To argue for a conceptual move from family-centred care (FCC) to a child-centred care approach and the implications for clinical nursing practice.	A child-centred care approach incorporates the rights of the child in all aspects of healthcare delivery in conjunction with the needs of their family. Key elements are protection, promotion and participation. A child-centred care approach requires the inclusion of the child’s perspective; a child’s needs must be considered in each situation and their rights to negotiate and choose is crucial. Children’s participation is a process that evolves over time and involves shared responsibility or negotiation of responsibility throughout childhood. CCC is underpinned by the concepts of trust, respect, autonomy and self-determination. A child-centred approach entails recognition and focusing on children’s agency and rights and the valuing of children’s voices, experiences and participation.
Coyne et al., 2018 Ireland, Sweden [4]	To identify the antecedents, attributes and relationship between family, person and child-centred care using a concept analysis.	Antecedents: The child is in the centre of thinking and practice. A child's perspective with joined participation and partnership, considering cultural and social aspects, strives for ethical symmetry, situated by using skills and strategies to recognise different ways of communication and listen to the child’s perspective. Attributes: Individualised own rights, dignity and respect, closeness with the family, social actor, own voice, consider competence and own engagement as an active agent. Consequences: The child's voice is heard, and each child’s competence and their own engagement as an active agent are respected.
Ford et al., 2018 Australia, New Zealand, UK [1]	To explore the concept of CCC and its potential theoretical alignment with an ecological approach to healthcare.	CCC has the potential to complement or extend traditional FCC, by placing children in a more prominent and central position than that which they currently hold within healthcare.
Foster 2015 New Zealand [30]	To propose a new paediatric model of care called family and child-centred care (FCCC).	An amalgamation of FCC and CCC needs to occur to create an FCC model, that includes both the characteristics of FCC and CCC, where the family and child are visible, at the forefront and equal in healthcare provision. This model then needs to be used by the government, organisations and institutions to plan, deliver and evaluate child healthcare provision.
Foster and Shields 2020 New Zealand, Australia [31]	To discuss different models of care for children and families and their components, philosophies and principles.	Core concepts of CCC (common to CFCC, PFCC and FIC) include respect, participation, partnership, information and consent. Core differences between the different approaches are whether the child, person and/or family as a unit are at the forefront. Agree that CCC occurs when ‘children and their interests need to be at the centre of our thinking and our practice, the inclusion of children and young people as active participants in their care’ (Carter et al., 2014). FCC and CCC are irrevocably interconnected and require a fluidic reciprocal interaction from both perspectives. Hence, a CFCC model is proposed by the authors.
Gerlach and Varcoe 2021 Canada [32]	To examine dominant discourses on CFCC in the context of families and children who are at greater risk of health inequities in wealthy countries.	Taking account of the growing recognition for socially responsive and inclusive healthcare approaches that mitigate the impacts of childhood adversity across the life course, there is an immediate need for research on how CFCC can be inclusive of and responsive to families and children who are vulnerable to health problems and healthcare inequities.
Lake, 2014 South Africa [33]	To share the lessons learned from delivering a short course in children’s rights and child law for health professionals in South Africa.	Integrating a child-rights approach into pre and in-service education provides a potentially powerful framework that nurses can draw on to give effect to children’s rights and legal entitlements, promote child health, improve quality, strengthen Intersectoral collaboration and an informed re-engineering of children’s services.
Shields, 2017 Australia [15]	To caution readers that we do not know what FCC is despite having used it for 30 years, and we need to understand CCC before we move to it.	Discussion draws on work by other authors. CCC views the child as the central person in healthcare interactions and children are active agents in their healthcare. They have the right to participate and need to be an integral part of partnerships in care. The family and parents remain central to the child’s health and well being. The child is an individual and their needs are paramount (Carter et al., 2014). Children are to be regarded as respected, singular agents who can represent and negotiate their own experiences and wishes (Coyne et al., 2016). Child-centred care (like FCC) sounds good, but it would be unethical to universally apply it to all children’s healthcare situations unless we know it works. International collaboration is needed to ensure a better understanding of the concept.
Söderbäck et al., 2011 Sweden, Ireland [23]	To discuss the differences between a child's perspective and the child’s perspective in healthcare settings.	No definition of CCC is presented, although the authors talk of a child-centred approach. Discussion on the child’s perspective includes features/principles of CCC. A FCC approach needs to be redirected toward a child-centred care approach that incorporates the rights of the child to participate in all aspects of healthcare delivery in conjunction with the needs of their family. The paper refers to Shier’s (2001) five-level model: irrespective of age, the child is listened to; the child is supported in expressing their views; the child’s views are taken into account; the child is involved in the decision-making process; and the child can share power and responsibility in the decision making.

**Table 2:** Empirical papers included in the review.

Author, Year, Country of Origin	Aim	Study Population	Intervention Type	Methodology	Level of Child Involvement	Important Results
Carlsson et al., 2021 Sweden [34]	To explore the impact of using an eHealth service (Sisom) to gain the children’s perspectives during their healthcare appointments.	Children (n = 16), aged 6–13 yrs, treated for different diseases.	The impact of using an eHealth service.	Constructivist grounded theory	Authentic	Implementing the use of Sisom (Norwegian acronym meaning ‘tell it how it is’) as a way to make children’s needs and preferences explicitly visible for decision making in practice and thereby supporting the further development of child-centred care in practice. The communication space thus enabled the children to voice their opinions on aspects of care which made the parents and the healthcare professionals listen to them and enabled a greater understanding and a higher level of participation for the children. Sisom can strengthen children’s empowerment and support the requirements for developing ways to make children’s needs and preferences explicitly visible in decision making in practice and thus support the ambition of furthering the development of child-centred care in practice.
Carnevale et al., 2017 Canada [35]	To examine how a relational ethics framework can improve clinical practice.	Children (n = 2), aged 24 yrs and 12 yrs.	None	Case study	Marginal	Conventional practices inadequately attend to the multiple ethical concerns encountered by these children, their families and the HCPs working with them. A relational ethics framework can promote clinical practices that are ethically attuned to the complexity of this population’s needs.
Carter, 2005 UK [36]	To explore the children’s/siblings’ perceptions of the (Salford) Diana Team.	Families (n = 5), involving children (n = 10), aged 2–13 yrs.	None	Qualitative participant inquiry	Authentic	The sick child’s siblings highlighted that attention to their needs was important. This study shows the value of including children in research about children’s services. Children use parents as their gold standard for care and they are clear about the skills and attributes they value about ‘outsiders’ who provide care to their family.
Castor, 2021 Sweden [37]	To describe nurses’ experiences of a child-centred family-guided intervention of obesity, targeting children identified as overweight and their caregivers.	Nurses (n = 13).	Child-centred family-guided interventions aiming to support families towards a healthier lifestyle.	Qualitative, descriptive inductive	Marginal	Emotional and practical challenges in performing CCHD still remained among nurses after customised training, which might include the child’s rights to be involved in their own care when the child was identified as overweight. Training for nurses, including lectures and tutorials, was found to increase the quality and professionalism of performing CCHD by providing structure, tools and tutorial support. Customised training and illustrations can support nurses when performing a structured intervention such as child-centred health dialogues.
Coombes et al., 2022 UK [38]		Children and young people (n = 26) aged 5–17 yrs, parents (n = 40), siblings (n = 13) aged 5–17 yrs, health and social care professionals (n = 15) and commissioners (n = 15).	None	Qualitative, inductive	Authentic	A child-centred approach to care needs to take an individual and holistic view of the child that ensures their physical, emotional, social, practical and spiritual needs are addressed. A child-centred approach to care for children with life-limiting conditions should incorporate support for the family, while ensuring the child remains the focus of care and their needs and interests are at the centre of care and decisions. Children as young as five wanted to be informed, supporting a child-centred approach where the child is, where able, an active participant.
Derwig et al., 2021 Sweden [39]	To test the feasibility of a Child-centred Health Dialogue model for primary prevention of obesity.	Children (n = 785); intervention (n = 203), control (n = 582).	Child-centred Health Dialogue	Non-randomised quasi-experimental cluster design	Authentic	This study demonstrates that a child-centred, multicomponent, interactive intervention for the promotion of healthy lifestyles and primary prevention of obesity for all 4-year-old children participating in Child Health Services is feasible on a small scale.
Derwig et al., 2021 Sweden [40]	To explore the experiences of children participating in CCHD.	Children (n = 21), aged 4 yrs.	Child-centred Health Dialogue (CCHD)	Qualitative, inductive	Authentic	4-year-old children given the opportunity to speak for themselves—elucidating the child’s perspective—interpreted the health messages in a different way than the intended meaning of the illustrations developed by adults. Findings are important for the improvement of CCHD and underline the utmost importance of including children in research on health promotion. 4-year-old children can take an active role in their health and are capable of making health information meaningful.
Foster and Whitehead, 2019 New Zealand, Australia [41]	To explore the lived experience of hospitalized school-aged children admitted to a paediatric high-dependency unit to gain insight into child-centred care.	Children (n = 26), aged 5–15 yrs.	None	Qualitative, interpretive phenomenological	Marginal	Defines CCC as when the child is central, at the forefront and the actor and co-constructor of care delivery within the context of the family and community. Core principles of CCC include the child being seen as a social being and a key agent in family partnerships and collaborations with staff where dignity, respect, honesty, privacy and opportunities to make decisions about their care are promoted. Children valued safety, respect, consultation, honesty, dignity, privacy and participation as key agents in family partnerships and collaborations with adults. Further research from a global and cultural perspective is required to understand the relationship between children, parents and staff, where communication, demography and health outcomes are explored from a CCC and FCC approach.
Gibbs et al., 2020 New Zealand [42]	To examine the lived experiences of nurses who care for children and their families admitted to hospital with a non-accidental head injury.	Nurses (n = 6).	None	Qualitative, hermeneutic phenomenological	Authentic	A child-centred approach places the child at the forefront of care, it recognises their rights to be recognised as active social agents and puts the child at the centre in relation to care planning. A child-centred approach does not negate the role of the family but positions the family differently in relation to being one of the many influencing ecological systems influencing the child’s health and well being (Ford et al., 2018).
Gondek et al., 2017 UK [43]	To review factors influencing person-centred care in mental health services for children, young people and families examining perspectives from professionals, service users and carers.	Papers (n = 23).	None	Systematic review	Marginal	The key recommendations of the review to improve provision of person-centred care are providing professionals with more training in using the approach, supporting them to use it flexibly to meet the unique needs of service users, whilst also being responsive to times when it may be less appropriate, and improving both the quantity and quality of information for service users.
Lipman et al., 2012 USA [44]	To learn how to serve families with children with diabetes in a more culturally effective manner.	Parents (n = 799).	None	Secondary data analysis	Marginal	There is a paucity of research on the goals and priorities of paediatric diabetes care from the perspective of parents from diverse racial backgrounds. Asking families about the type of care they prefer may help to improve the design and delivery of services in a culturally competent, effective manner.

## Data Availability

No new data were created.

## Supplementary Materials

The following supporting information can be downloaded at: https://www.mdpi.com/article/10.3390/pediatric16010012/s1.
