# Supplementary material for: The Concept of Child-Centred Care in Healthcare: A Scoping Review
Source: Pediatr Rep. 2024 Feb 1;16(1):114–34. doi: 10.3390/pediatric16010012 (PMC10885088; doi:10.3390/pediatric16010012)
Supplement: Supplementary file 1 [file pediatrrep-16-00012-s001.zip › pediatrrep-2712713-supplementary.pdf]

Search Terms and Strategies

1. **CINAHL**

Search ID#	Search Terms
S10	S6 AND S7
S9	S6 AND S7
S8	S6 AND S7
S7	S3 OR S4 OR S5
S6	S1 OR S2
S5	MH child* cent*ed care OR child health services
S4	MH child* cent*ed care OR family cent*ed care
S3	MM patient autonomy OR decision making, family
S2	AB child* cent*ed OR child cent*ed care OR child health care OR child* decision-making
S1	TI child* cent*ed OR child* cent*ed care OR child* health care OR child* decision-making

Close

2. Web of Science

8	**#5 AND #6** and **English** (Languages)
7	**#5 AND #6**
6	**#3 AND #4**
5	**#1 AND #2**
4	**child health services** (Abstract)
3	**patient autonomy OR decision making, family** (Abstract)
2	**child* cent*ed OR child* cent*ed care OR child* health care OR child* decision-making** (Abstract)
1	**child* cent*ed OR child* cent*ed care OR child* health care OR child* decision-making** (Title)

3. Medline

S8	S6 AND S7
S7	S3 OR S4 OR S5
S6	S1 OR S2
S5	MH child* cent*ed care OR child health services
S4	MH child* cent*ed care OR family cent*ed care
S3	MM patient autonomy OR decision making, family
S2	AB child* cent*ed OR child cent*ed care OR child health care OR child* decision-making
S1	TI child* cent*ed OR child* cent*ed care OR child* health care OR child* decision-making
